# Effect of Preoperative Anxiety on Depth of Anaesthesia and In Vitro Fertilization Success

**DOI:** 10.4274/TJAR.2023.22829

**Published:** 2023-10-24

**Authors:** Sevtap Hekimoğlu Şahin, Elif Çopuroğlu, Ece Yamak Altınpulluk, Necdet Süt, Beyhan Karamanlıoğlu, Koray Elter, Özge Yaman

**Affiliations:** 1 Department of Anaesthesiology and Reanimation, Trakya University Faculty of Medicine, Edirne, Turkey; 2 Morphological Madrid Research Center (MoMaRC), UltraDissection Spain EchoTraining School, Madrid, Spain; Outcomes Research Consortium, Cleveland, Ohio, USA; Anaesthesiology Clinical Research Office, Atatürk University, Erzurum, Turkey; 3 Department of Bioistatistics, Trakya University Faculty of Medicine, Edirne, Turkey; 4 Department of Obstetrics and Gynecology, Trakya University Faculty of Medicine, Edirne, Turkey

**Keywords:** Anxiety, depth of anaesthesia, monitoring of brain function, oocyte retrieval, sedation

## Abstract

**Objective:**

Infertility anxiety may have a harmful effect on embryo quality and fertilization during in vitro fertilization (IVF). Monitoring brain function gives real-time information about the depth of anaesthesia of a patient. This study examined the effect of preoperative anxiety on the depth of anaesthesia and IVF success.

**Methods:**

One hundred thirty-one patients who had undergone oocyte retrieval were divided into two groups according to the Beck Anxiety Inventory (BAI): the low-anxious Group L (n = 71) and high-anxious Group H (n = 60). Hemodynamic stability, intraoperative total propofol and fentanyl consumption, good quality embryo (GQE) rate, and fertilization rate were recorded.

**Results:**

Fertilization and GQE rates were not significant between groups L and H. Total propofol consumption was significantly higher in group H than in group L. Heart rate (HR) preoperatively and postoperatively and systolic arterial pressure (SAP) preoperatively and diastolic arterial pressure (DAP) postoperatively were significantly increased in group H than in group L. The time for the modified Aldrete score to reach 9 (MAS 9) in group H was significantly higher than that in group L. The effect of variables that were found significantly in the univariate analysis (Propofol, HRpreop, HRpostop, SAPpreop, DAPpostop, and MAS 9) on BAI score.

**Conclusion:**

Total propofol consumption was higher in patients with high anxiety levels, but it did not have a negative effect on IVF success.

**Main Points**

• Infertility is usually accompanied by psychological and behavioral changes and can result in postoperative anxiety.

• Monitoring brain function gives real-time information about the depth of anaesthesia of a patient.

• The increased total anaesthetic drug consumption under monitoring brain function does not have any negative effect on the fertilization rate, embryo quality, and/or pregnancy rate.

## Introduction

Anxiety is common in women undergoing infertility treatment. Psychological and behavioral changes can often accompany infertility and cause pre-operative anxiety.^1^ Therefore, when these women are hospitalized for oocyte retrieval, the degree of anxiety can be much higher. The absence of premedication before oocyte retrieval may further increase anxiety. In addition, this situation can negatively affect the total consumption of analgesics and anaesthetic drugs and recovery from anaesthesia. The impact of anaesthetic agents on embryo quality and fertilization has not been clearly definite until today. Previous studies have described different results concerning the negative effects of anaesthetic drugs on fertilization and embryo development.^2,3,4,5,6,7^

Propofol is an excellent drug for outpatient surgery. The level of propofol that is spread in follicular fluid is indicated to increase in proportion to the total dose of propofol consumed.^2,4^ Studies examining the effect of propofol on in vitro fertilization (IVF) technologies have reported conflicting results during the studies.^8,9^

Opiates are generally used with propofol for sedation during oocyte retrieval. Soussis et al.^6^ reported low follicular fluid concentrations of fentanyl and alfentanil during transvaginal oocyte retrieval in patients, but they did not find any difference between the groups in terms of fertilization rate or pregnancy rate. In a study, general anaesthesia versus sedation with remifentanil for assisted reproduction was compared, and the pregnancy rate in the group under general anaesthesia was found to be significantly lower.^10^

Monitoring brain function gives real-time information about the depth of anaesthesia of a patient. Brain function monitoring helps reduce anxiety by prevention awareness and the harmful effects of a low dose of anaesthesia. Previous studies have demonstrated the effects of many factors on pregnancy rates and oocyte number during IVF treatment.^11,12^ However, the effect of anxiety level with anaesthetic drugs on embryo and oocyte quality during IVF in women undergoing sedation has not yet been studied. We aimed to examine the effect of preoperative anxiety on depth of anaesthesia, embryo quality, fertilization, and pregnancy rates.

## Methods

This prospective, single-center, double-blind study was approved by the local ethical committee, was conducted in patients who underwent surgery at Trakya University (ClinicalTrials.gov Identifier: NCT03134651). The patients were informed about the study and their written approval was obtained. A total of 131 adult patients with American Society of Anesthesiologists physical status I-II, aged 25-43 years, and who were scheduled for oocyte retrieval under sedation were enrolled in this study. The exclusion criteria were; (1) unable to communicate well in the native language, (2) secondary infertility can be surgically corrected, (3) history of psychiatric illness, (4) women who necessitated general anaesthesia.

### Clinical Evaluation and the Method

The anaesthesiologists evaluated all patients the day before the surgery at the surgical clinic. Patient characteristics (age, body mass index, duration of surgery, smoker, alcohol, reason for infertility, etc.) were recorded. Beck’s Anxiety Inventory (BAI) consists of questions concerning 21 symptoms of cognitive and somatic anxiety. The responses of the patients were rated on a scale from 0 to 3, and the highest score was 63. The validity and reliability of this test translated into the Turkish has been approved by Ulusoy et al.^13^ The cut-off score of 17 was determined for BAI. All data from the study were gathered by two anaesthesiologists. The first anaesthetist evaluated the BAI score in patients and recorded the results. Patients were separated into two groups according to the pre- procedure BAI score: Group L (low-anxiety group) and Group H (the high-anxiety group). The BAI scores of the low-anxiety group are equal to or less than 17 and those of the high-anxiety group are more than 17.^13,14^ Intraoperative and postoperative data were recorded by a second anaesthetist.

All women in this study were fasting for 8 h and none received premedication (opioid, antiemetic, sedative). Electrocardiogram, pulse oximetry (SpO_2_ in %), non-invasive blood pressure, axillary temperature (T), and end-tidal carbon dioxide were used for standard monitoring in the operating room. The Patient State Index (PSI) SEDLine (Masimo Inc., California, USA) sensor was placed simultaneously with other standard monitors before the induction of anaesthesia. In all women, pre-oxygenation was administered via face mask during the procedure. After preoxygenation, all patients received 1.5 mcg kg^-1^ fentanyl and a 2-3 mg kg^-1^ propofol bolus for induction. Anaesthesia was maintained with an infusion of 150 µg kg^-1^ min propofol. The anaesthesiologist applied 0.5-1.0 mg kg^-1^ propofol boluses to keep PSI values between 40 and 60. Respiration was supported by manual ventilation or oxygenation during the procedure.

PSI, heart rate (HR), SpO2, diastolic arterial pressure (DAP), and systolic arterial pressure (SAP) were recorded at baseline, 15 min, and postoperatively. The postoperative sedation score was evaluated using the Ramsay Sedation Scale (RSS). At the end of the process, the operation time and total propofol and fentanyl consumption were recorded.

Propofol infusion was discontinued 5 min before the completion of the surgical procedure. The patients were sent to the recovery unit after spontaneous breathing and cognitive functions were assessed. Nausea, vomiting, agitation, and tremors were recorded by an independent investigator throughout the procedure.

The modified Aldrete score (MAS) was used to assess patients’ recovery from anaesthesia. These parameters are rated on a scale from 0 to 2. MAS was recorded every 3 min in the recovery unit. When the MAS was equal to or more than 9, patients were transferred to the clinic. The time for MAS to reach 9 was recorded. Patients were assessed for pain using the visual analog scale (VAS) at 1, 2, and 4 h postoperatively by an anaesthesiologist who was not included in this study. Oral acetaminophen at 500 mg was used as a rescue analgesic when VAS scores were more than 4 in each of the two groups within 4 h.

### Ovarian Stimulation

Antagonist cycles were performed in all women. When adequate stimulation was achieved, human chorionic gonadotropin was administered. The oocyte retrieval procedure was performed using a single lumen aspiration needle (Reproline, Rheinbach, Germany). All cycles were intracytoplasmic sperm injection cycles. After 2-5 days, one or two embryos were transferred. A pregnancy test was performed on the 12^th^ day after embryo transfer. Clinical pregnancy was evaluated and confirmed by ultrasound, 3 weeks after embryo transfer.

In this study, the fertilization rate and good quality embryo (GQE) rate were investigated in patients who had undergone oocyte retrieval. The fertilization rate was described as follows: number of fertilized oocytes (=2 pronuclei, PN)/The number of retrieved oocytes. The GQE rate was calculated as follows: the number of GQEs/The number of 2 PN zygotes. A GQE was described as having one to four to six cells on the 2^nd^ day, 6-10 cells on the 3^rd^ day with <20% fragmentation and no multinucleation, and finally a tightly packed inner cell mass and trophectoderm cells in a cohesive layer on the 5^th^ day.^7^ The fertilization and GQE rates were calculated per patient. Pregnancy rate was the secondary outcome.

### Statistical Analysis

The sample size was 64 patients in each group to detect a middle effect size (d=0.5) in the number of oocytes retrieved between the high and low BAI score groups with an alpha level of 5% and power of 80%. The normality distribution of all numeric variables was tested using the one-sample Kolmogorov-Smirnov test. Variables that were normally distributed between the high and low BAI score groups were compared using Student’s t-test. Categorical data were compared using the chi-square test. Variables that were non-normally distributed between the high and low BAI score groups were compared using the Mann-Whitney U test. The effect of variables that were found to be significant in the univariate analysis (Propofol, HR_preop_, HR_postop_, SAP_preop_, DAP_postop_, and MAS 9) on BAI score was investigated using multivariate logistic regression analysis. A *P* value of 0.05 was set as statistically significant.

## Results

**Figure 1:**
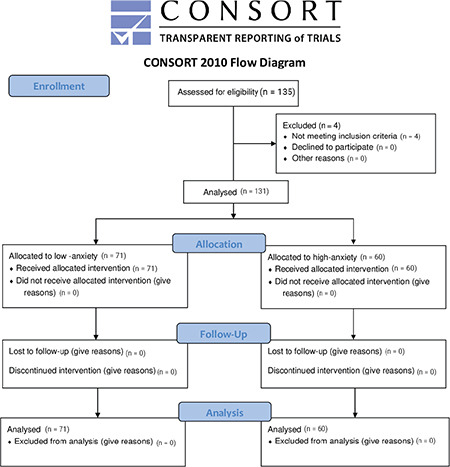
Flow diagram.

One hundred thirty-five patients were included in this study, four patients dropped out because of the necessity of general anaesthesia. Therefore, 131 patients were analyzed according to the protocol (Figure 1). There was no statistically significant difference between the groups with respect to body mass index, age, duration of surgery, and patient characteristics (Table 1).

HR preoperatively and postoperatively and SAP preoperatively and DAP postoperatively were significantly increased in group H than in group L (*P*=0.002, *P *< 0.046, *P *< 0.040, and *P *< 0.025, respectively). PSI values and SpO_2_ preoperatively were similar in groups L and H.

Total propofol consumption was significantly higher in group H than in group L (*P*=0.006). Total fentanyl consumption and VAS scores were not significantly different between the groups (Table 2).

MAS 9 was significantly increased in group H than in group L (*P* < 0.001). Postoperative RSS was not significant between groups (Table 3). The side effects are presented in Table 3. The groups were similar with respect to nausea, agitation, and shivering. None of the patients in the two groups suffered other side effects (rash, dizziness, headache, or allergic reaction).

The effects of propofol, HR_preop_, HR_postop_, SAP_preop_, DAP_postop_, and the time for MAS to reach 9 on the BAI score were investigated through multivariate logistic regression analysis. HR_preop_ and MAS to reach 9 were found to be related factors with a high BAI score (*P*=0.020 and *P*=0.001, respectively). According to the results of the multivariate logistic regression model, when HR_preop_ [odds ratio (OR)=1.049: 95% confidence interval (CI): 1.008-1.092] and MAS reach time 9 (OR=1.503; 95% CI: 1.179-1.916) increases the risk of high BAI score increases.

The fertilization and GQE rates were not significantly different between groups L and H (*P*=0.848, *P*=0.349, respectively). The mean number of embryos transferred, day of embryo transfer, and pregnancy rate was similar between the groups (Table 4).

## Discussion

The most valuable result of this study was that the increased total propofol consumption in the high-anxiety group did not have any negative effect on the fertilization rate, embryo quality, and/or pregnancy rate. Moreover, MAS 9 was significantly higher in group H than in group L.

Anaesthetic management is very difficult in women with high levels of anxiety due to the lack of any premedication. The absence of premedication, due to significant surgical stimulation during needle insertion and prevention of awareness, may require increased total consumption of analgesic and anaesthetic drugs and deeper levels of sedation to provide optimal patient comfort and surgical status.^15^ Patients undergoing brain function monitoring can be protected from the harmful effects of over- or low-dose anaesthesia.

Oocyte retrieval is generally performed using sedation in an ambulatory setting and is a short operation. There are many studies investigating the effects of various anaesthetic drugs used on fertilization and embryo quality or pregnancy rate during oocyte retrieval.^16,17,18,19^ In animal studies in mice, exposure of mouse oocytes to propofol caused toxic effects on fertilization.^20,21^ Human studies have reported conflicting results regarding the side effects of anaesthetic agents on fertilization and embryo quality.^7,22,23^ Conscious sedation and general anaesthesia are well tolerated by propofol, opioids, benzodiazepines, nitrous oxide, or other drugs for women and oocytes, but further studies are needed to find the ideal drug or technique combination for women and oocytes.^24^ In our study, we did not observe any negative effects on fertilization and embryo quality or the pregnancy rate in women who required high-doses of propofol because of preoperative anxiety. Unlike our study, Wilhelm et al.^10^  retrospectively compared the effects of remifentanil versus general anaesthesia during oocyte retrieval on fertility and embryo quality in 251 women. In this study, general anaesthesia induction was performed using propofol and nitrous oxide and was maintained with isoflurane or a propofol infusion. In the other group, all patients received standardized under monitored anaesthesia care with remifentanil infusion, but local anaesthetics were not used. They reported that the rate of pregnancy in the general anaesthesia group was significantly lower. Christiaens et al.^4^ reported that the time-dependent diffusion and accumulation of propofol in follicular fluid were related to the dose of propofol used. They suggested that the total dose of propofol administered during anaesthesia should be limited. A study by Coetsier et al.^2^ supports these informations, and furthermore, they reported higher blood and follicular fluid concentrations of propofol because of the administration of a smaller dose of alfentanil. They recommend that the oocyte retrieval procedure should be as short as possible to reduce anaesthetic drug accumulation in the follicular fluid. Although the total amount of propofol consumed in these studies was not reported, the protocol for propofol sedation was similar to ours. In our study, total propofol consumption was significantly higher in the high-anxiety group 217.8 (37.3) than in the low-anxiety group 199 (32.8). Nevertheless, these values were not considered to be clinically meaningful as neither the fertilization rate nor the GQE rate were found to be significantly different between the groups.

There are studies investigating the effect of emotional anxiety on reproductive success in infertile women without evaluating anaesthesia.^1,25,26^ Anderheim et al.^26^ investigated the effect of psychological stress on IVF outcomes before and during IVF treatment. They obtained no evidence that psychological stress had an effect on the IVF outcome. The authors did not report any anaesthetic drugs used in this study. To the best of our knowledge, the effect of preoperative anxiety on depth of anaesthesia and IVF success has not been compared during oocyte retrieval. In this study, reduced total propofol consumption in the low-anxiety group did not increase fertility success. However, the time for anaesthesia recovery was significantly increased in the high-anxiety group. In addition, brain function monitoring could be helpful in preventing awareness and preventing the harmful effects of an overdose of anaesthesia. Sedation depth is important for preventing anxiety. We believe that brain function monitoring in our study is important in assessing the validity of our data.

Serious side effects related to propofol and fentanyl were not observed during oocyte retrieval in our study. The high and low anxiety groups were clinically similar with respect to hemodynamic state, postoperative VAS scores for pain, nausea, and vomiting. However, the time for anaesthesia recovery increased in the group with high anxiety.

### Study Limitations

There are several factors that limit this study. First, the results of our study cannot be generalized to other anaesthesia techniques. Second, oocyte retrieval is not performed without anaesthesia for comparison. Third, this study is important at first in assessing embryo quality but second in assessing the pregnancy rate because many factors influence the pregnancy rate after sedation. Fortunately, the two groups in this study detected homogeneous results relating to gender, age, and ASA preoperatively.

## Conclusion

In conclusion, preoperative anxiety can commonly be observed before oocyte retrieval. In this study, it can be said that propofol and fentanyl can safely be administered with monitoring brain function to prevent preoperative anxiety during oocyte retrieval, so high level of anxiety will not have negative effect on embryo quality, fertilization, and pregnancy rates.

## Figures and Tables

**Table 1:**
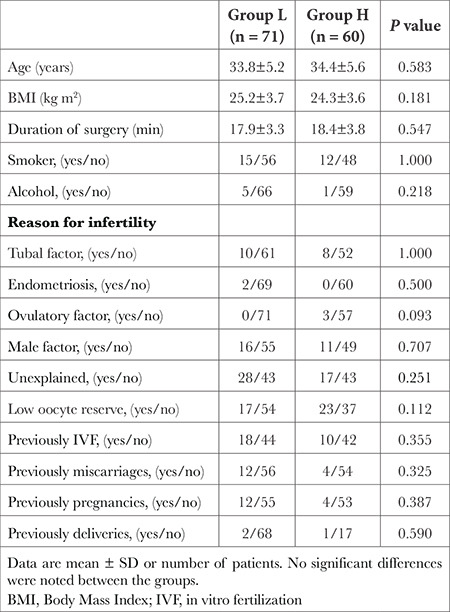
Demographic, Surgery Data and Patient Characteristics

**Table 2:**
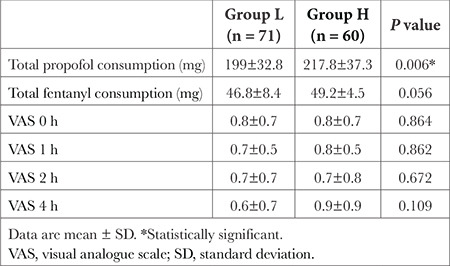
Total Intraoperative Propofol and Fentanyl Consumption and Post-Operative VAS Scores

**Table 3:**
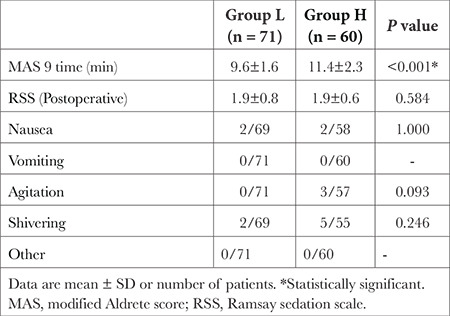
Postoperative Recovery and Side Effects by Groups

**Table 4:**
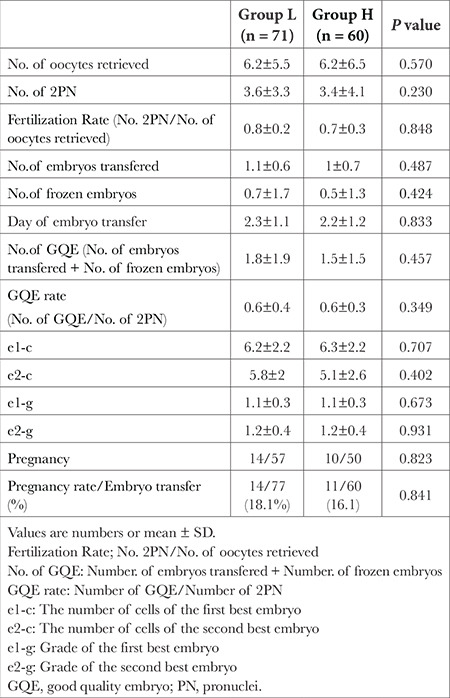
Oocyte and Embryo Quality by Groups
